# Advances on diagnostic biomarkers of pancreatic ductal adenocarcinoma: A systems biology perspective

**DOI:** 10.1016/j.csbj.2020.11.018

**Published:** 2020-11-21

**Authors:** Wu-Hu Zhang, Wen-Quan Wang, Xuan Han, He-Li Gao, Tian-Jiao Li, Shuai-Shuai Xu, Shuo Li, Hua-Xiang Xu, Hao Li, Long-Yun Ye, Xuan Lin, Chun-Tao Wu, Jiang Long, Xian-Jun Yu, Liang Liu

**Affiliations:** a Department of Pancreatic Surgery, Fudan University Shanghai Cancer Center, Shanghai, China; b Department of Oncology, Shanghai Medical College, Fudan University, Shanghai, China; c Shanghai Pancreatic Cancer Institute, Shanghai, China; d Pancreatic Cancer Institute, Fudan University, Shanghai, China

**Keywords:** Pancreatic ductal adenocarcinoma, Systems biology, Genomics, Transcriptomics, Proteomics, Bioinformatics

## Abstract

Pancreatic ductal adenocarcinoma (PDAC) is a lethal malignancy that is usually diagnosed at an advanced stage when curative surgery is no longer an option. Robust diagnostic biomarkers with high sensitivity and specificity for early detection are urgently needed. Systems biology provides a powerful tool for understanding diseases and solving challenging biological problems, allowing biomarkers to be identified and quantified with increasing accuracy, sensitivity, and comprehensiveness. Here, we present a comprehensive overview of efforts to identify biomarkers of PDAC using genomics, transcriptomics, proteomics, metabonomics, and bioinformatics. Systems biology perspective provides a crucial “network” to integrate multi-omics approaches to biomarker identification, shedding additional light on early PDAC detection.

## 1 Introduction

Pancreatic ductal adenocarcinoma (PDAC) is an aggressive cancer of the digestive system with increasing incidence and very high mortality [1]. Despite significant improvements in the diagnosis and treatment of PDAC in the last few decades, PDAC is the third most common cause of cancer-related deaths and will become the second leading cause behind lung cancer in the United States by 2030 [2]. Most patients with PDAC are diagnosed at advanced stages when curative surgery is no longer possible. Accordingly, outcomes for patients with PDAC are always poor. Robust biomarkers with high sensitivity and specificity for early detection would enable curative resection of PDAC, reducing the high mortality rate. Therefore, the development of early diagnostic PDAC biomarkers is an urgent clinical concern.

Carbohydrate antigen 19-9 (CA19-9) is currently the only biomarker approved for clinical PDAC diagnosis; however, it is insufficient as an independent diagnostic tool, because it has only 50–75% sensitivity and 83% specificity in symptomatic patients, which can lead to false-positive results and misdiagnoses [3], [4], [5]. For instance, serum CA19-9 elevation can be found in patients with benign diseases such as chronic or acute pancreatitis, cholangitis, obstructive jaundice, liver cirrhosis, or other malignancies, such as gastrointestinal cancers [6]. Additionally, about 13% of patients with PDAC do not have CA19-9 elevation [7]. Therefore, biomarkers with higher sensitivity and specificity are needed.

**Fig. 1:**
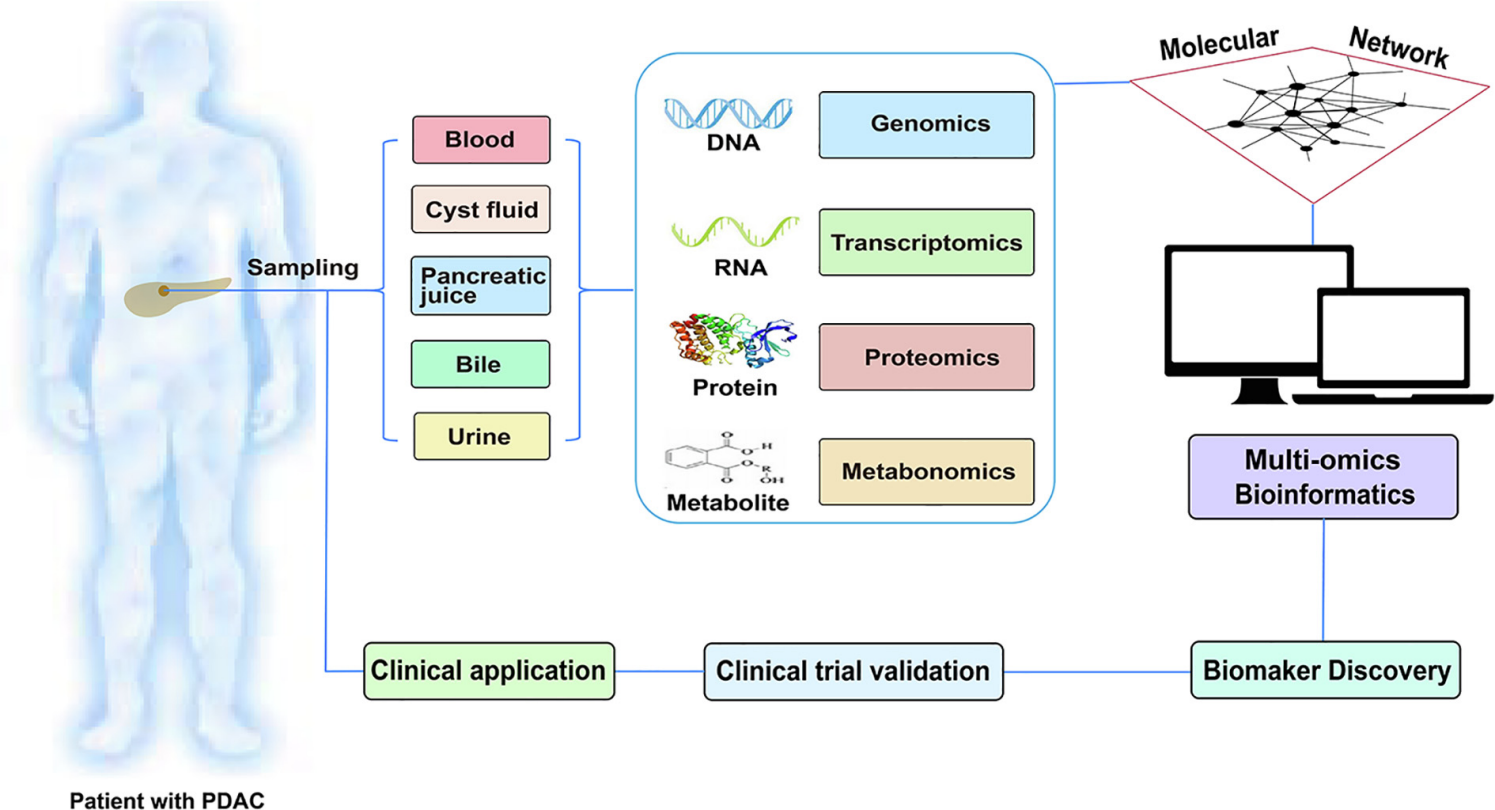
A systems biology overview of research on diagnostic biomarkers for early detection of pancreatic ductal adenocarcinoma (PDAC). Sampling sources including blood, cyst fluid, pancreatic juice, bile, and urine can be used for biomarker analysis. Systems biology integrates genomics, transcriptomics, proteomics, and metabonomics data into an integrated molecular network. Bioinformatics tools are applied to the integrated data to discover diagnostic biomarkers. Once the biomarkers are verified in clinical trials, they may eventually be applied in clinical practice.

Systems biology perspective is a holistic and collaborative approach that can be considered as a “network” that integrates experiment, theory, and quantitative modelling [8], [9]. Using system biology as a network, it is possible to study complex medical concerns in an organized and integrated way rather than piecemeal using different approaches separately. The systems biology approach combines genomics, transcriptomics, proteomics, and metabonomics to allow identification and quantification of molecules with increasing accuracy, sensitivity, and comprehensiveness. Recent independent studies have reported potential biomarkers for early PDAC diagnosis; however, those findings have yet to be integrated from a systems biology perspective. In this review, we present a systems-level outlook on investigations of diagnostic biomarkers of PDAC (Fig. 1).

## 2 Genomics

Large-scale genomics studies and genomic techniques such as next-generation sequencing (NGS) provide great potential to assist early diagnosis and screening of PDAC. Individuals who carry alleles that predispose them to cancer development will benefit from early detection by genomics methods.

The development of NGS led to the identification of various potential biomarkers including chromosomal rearrangements, driver mutations, gene expression changes, single nucleotide polymorphisms (SNPs), and copy-number variations; however, no definitive markers have been specified for PDAC [10]. Norris and colleagues performed a comprehensive genomics analysis to compare four major PDAC driver genes (*KRAS*, *CDKN2A*, *TP53*, and *SMAD4/DPC4*) in familial and sporadic PDAC using whole-exome sequencing, whole-genome sequencing (WGS), RNA sequencing, and high-density SNP microarrays [11]. They concluded that the four major driver genes had the same potential utility for early diagnosis in familial PDAC and sporadic PDAC. Hu et al. conducted the largest study to date of inherited germline mutations in PDAC using targeted sequencing [12]. They determined that six cancer-predisposition genes (*CDKN2A*, *TP53*, *MLH1*, *BRCA2*, *ATM*, and *BRCA1*) were associated with increased risk of PDAC. On the basis of those results, Hu et al. suggest that it is time to consider the use of genomics to enrich high-risk individuals and screen for inherited PDAC susceptibility, because personal and family history alone cannot reliably identify the majority of individuals with elevated risk.

PADC is genetically diverse, with tumors commonly harboring more than 60 genetic alterations [13]. Although a large number of novel gene mutations and genetic aberrations have been found in PDAC, it is difficult to know what impact genomic variables have on the transcriptome, proteome, and metabonome of cells, especially when genetic mutations are localized in non-coding regions [14]. Additionally, the high cost and lack of standardization of NGS techniques limit the use of genomics for cancer biomarker discovery. Hence, genomics methods can help to identify high-risk individuals and make early diagnoses of PDAC, but their value might be limited in the absence of corresponding multi-omics data [14].

Circulating tumor cells (CTCs) are cells that are derived from primary tumors or metastatic sites and circulate in the bloodstream. Rhim et al. reported the potential diagnostic role of CTCs using a geometrically enhanced differential immunocapture chip [15]. They showed that CTCs were detectable in 73% of patients with PDAC and 0% of healthy controls and, intriguingly, that circulating pancreatic epithelial cells could be detected before tumors became visible [15]. CTCs have shown high sensitivity (>70%) for detecting early-stage PDAC and are therefore regarded as a promising biomarker [16], [17]. Analyses of CTCs need to not only determine CTC-specific genomic alterations with NGS techniques but also use proteomics, transcriptomics, and metabonomics to identify other CTC-specific signatures. For example, Abouleila et al. used living single-cell mass spectrometry to demonstrate that single CTCs had different metabonomic profiles corresponding to different types of organ-specific tumors [18]. CTCs might be a good source of biomarkers for early PDAC diagnosis, but some limitations need to be addressed. First, CTCs are rare and heterogeneous, and it is still a challenge to detect them accurately [19]. Second, a standard methodology and large-scale validations are urgently needed to enable wider clinical use of CTCs [20].

Cell-free circulating tumor DNA (ctDNA) refers to small fragments of DNA that are released from cancer cells and contain characteristic tumor information. The detection of ctDNA by NGS techniques might offer an easily accessible and non-invasive way to diagnose PDAC [21]. Cohen et al. reported that combined analyses of ctDNA and protein biomarkers could improve the sensitivity while retaining specificity for early detection of PDAC compared with analysis of protein biomarkers alone [22]. In addition, ctDNA provides a useful tool to differentiate between malignant intraductal papillary mucinous neoplasm (IPMN) and other harmless pancreatic tumors. Berger et al. reported that *GNAS*-mutant DNA was present in the plasma of 71.4% of patients with IPMN but not in that of healthy individuals or patients with serous cyst adenoma [23]. A joint review by the American Society of Clinical Oncology and the College of American Pathologists concluded, however, that the clinical validity and utility of ctDNA for the early diagnosis of cancer were not yet sufficiently supported by evidence, and further research was warranted [24].

In addition to ctDNA, epigenetic biomarkers such as DNA methylation, histone modification, and chromatin structure might improve the early detection of PDAC [25]. For example, Yi and colleagues reported that DNA methylation in the promoters of *BNC1* and *ADAMTS1* was a promising marker to detect early-stage PDAC, with an overall sensitivity of 81% and a specificity of 85% [26]. Similarly, Elissa et al. showed that the promoter methylation status of *ADAMTS1* and *BNC1* could serve as a biomarker for early diagnosis of PDAC (stages I and II), with the two-gene panel showing a sensitivity of 94.8% and a specificity of 91.6% [27].

It is a challenge to develop epigenetic biomarker panels in asymptomatic populations because of the rarity and heterogeneity of PDAC. Although genome-wide assays are rapidly creating datasets of gene expression changes, ctDNA, and epigenetic biomarkers, challenges and difficulties remain for the development of diagnostic biomarkers of PDAC. Therefore, a systematic approach that integrates multi-omics studies is warranted, and genomic data should be combined with transcriptomics, proteomics, metabonomics, and clinical characteristics to facilitate the development of diagnostic biomarker signatures.

## 3 Transcriptomics

Transcriptomics studies are generally performed using RNA sequencing (RNA-seq) [28], quantitative real-time PCR (qPCR), or microarrays [29]. The most common application of transcriptomics in PDAC research is to compare gene expression between tumors and normal pancreas tissues to provide catalogs of transcripts that show altered expression in tumors. Such datasets can be used to identify individual transcripts that show large changes in tumors, or to create an overall map or ‘signature’ of multiple expression changes that are associated with tumors [30]. In addition, non-coding RNAs, including microRNAs (miRs) and long non-coding RNAs (lncRNAs), play important regulatory functions [31], [32]. Critically, transcription at each step of tumor development can be quantitatively assessed, providing opportunities for non-invasive and early diagnosis of PDAC.

Wang and collaborators performed miR profiling of pancreatic juice and found that the combination of miR-205, miR-210, miR-492, and miR-1427 could predict PDAC with a sensitivity of 87% and a specificity of 88% [33]. Müller et al. used RNA-seq to show that several RNAs were differentially expressed between six PDACs and five normal pancreas tissues, including miRs (miR-802, miR-2114, or miR-561), snoRNA-derived RNAs such as sno-HBII-296B and a piwi-interacting RNA (piR-017061), long intergenic non-coding RNAs (lincRNAs; LINC00261 and LINC00152), and natural antisense transcripts (HNF1A-AS1 and AFAP1-AS1) [31]. Vila-Navarro and colleagues conducted a miRNAome analysis of PDAC, IPMN, and healthy control tissues [34]. They identified and validated 30 miRNA-based biomarkers to discriminate PDAC or IPMN from healthy control tissues in two independent cohorts. Remarkably, miR-93, miR-16, miR-548d-3p, and miR-320a presented high discriminative accuracy for PDAC in endoscopic ultrasound-guided fine needle aspirations, with areas under receiver-operating characteristic curves (AUCs) of >0.95. Liu et al. analyzed bioinformatics databases and reported that the circulating lncRNA ABHD11-AS1 was a potential biomarker for early detection of PDAC with a sensitivity of 89.4% and a specificity of 88.6% [35]. Zhou et al. identified a signature of seven lncRNAs for early detection for PDAC with a sensitivity of 72.2% and a specificity of 33.3% [36]. They developed a novel diagnostic tool called LncRisk-7 based on the expression of the seven lncRNAs, which achieved high performance diagnosing PDAC in a discovery cohort and two independent validation cohorts [36]. Although non-coding RNAs have displayed diagnostic value for the early detection of PDAC, the application of miRs or lncRNAs as biomarkers of PDAC remains relatively rare.

Single-cell transcriptomics might facilitate the development of molecular biomarkers for early diagnosis. Bernard et al. used single-cell RNA-seq to reveal how the tumor microenvironment evolves during the neoplastic progression of IPMN to PDAC, providing unparalleled insight into early cancer pathogenesis [37]. Their analysis of single-cell transcriptomes over the course of PDAC progression demonstrated gradual depletion of proinflammatory immune components such as CD8^+^ cytotoxic T cells, CD4^+^ activated helper T cells, and dendritic cells, accompanied by infiltration of myeloid-derived suppressor cells and heterogeneous stromal myofibroblast populations.

Overall, it is clear that rapidly advancing techniques for transcriptomics will have a continuous impact on biomarker studies for years to come, but challenges remain. First, transcriptomics studies involve extensive sample preparation, high financial cost, and enormous computational requirements to handle the large amounts of sequence data. Second, RNA-seq is fruitless if the potential pathogenic variant does not have altered RNA abundance or sequence, in which case proteomics may come into play [38].

## 4 Proteomics

Proteomics is the study of a complete set of expressed proteins in terms of their localization, functions, post-translational modifications, and protein–protein interactions [39]. At the clinical level, cancer-related proteins or peptides in body fluids might provide information for the early detection of PDAC. The continuously improving sensitivity and high reliability of mass spectrometry (MS)-based proteomics play an important role in the discovery and validation of novel protein biomarkers.

Shalini Makawita et al. demonstrated islet-derived 1 beta (REG1B) and syncollin (SYCN) as candidate biomarkers of PDAC in 2011 and 2013, respectively, by using two-dimensional liquid chromatography/tandem mass spectrometry (LC-MS/MS) for proteomic analysis of six pancreatic juice samples from patients with PDAC and conditioned media from six pancreatic cancer cell lines and one normal pancreatic ductal epithelial cell line, followed by further validation of the two biomarkers by ELISA [40], [41]. In 2016, Sogawa et al. performed a quantitative proteomics analysis using LC-MS/MS and reported that complement component 4 binding protein α-chain (C4BPA) could be used as a serum biomarker for early PDAC detection and for differentiation between PDAC and other gastroenterological cancers [42]. In addition, Guo et al. performed MS proteomic profiling to screen for serum biomarkers of PDAC and identified dysbindin as a potential diagnostic biomarker to discriminate PDAC from chronic pancreatitis with a sensitivity of 81.9% and a specificity of 84.7% [43]. Those authors further showed that dysbindin maintained its high diagnostic accuracy in patients with PDAC who were negative for CA19-9 elevation. Furthermore, Ligat et al. reported distinct plasma peptide patterns between benign and advanced PanIN lesions, demonstrating the feasibility of developing novel biomarkers for early detection of PDAC by proteome profiling [44].

Root et al. nominated four key studies of diagnostic protein PDAC biomarkers, which, although subjective, reported that panels including CA19-9 and other markers outperformed CA19-9 alone [45]. Cohen et al. reported that ctDNA testing combined with testing of four plasma proteins (CA19-9, CEA, hepatocyte growth factor, and osteopontin) outperformed CA19-9 testing alone to differentiate PDAC from healthy controls, pancreatitis, and other benign diseases [22]. In another study, Capello et al. showed that the combination of TIMP1, LRG1, and CA19-9 outperformed CA19-9 alone [46]. Additionally, Kaur et al. surveyed the literature and found that MUC5AC was a favorable biomarker of PDAC that, when combined with CA19-9, outperformed CA19-9 alone [47]. Kim et al. reported that a two-biomarker panel of thrombospondin (THBS)-2 and CA19-9 outperformed CA19-9 alone [48].

Proteomics has deepened our understanding of the biology of PDAC and has great potential to discover novel diagnostic biomarkers for early detection of PDAC. It is necessary to verify the most promising biomarkers in large patient cohorts and, when justified, accelerate their clinical use. Several limitations and clinical challenges remain, however. First, the proteome of tissues and cells is dynamic, and proteomic evaluation at any given time only shows the current state of the cells [14]. Second, the forms and functions of proteins vary because of alternative splicing and post-translational modifications, presenting additional challenges to proteomic analysis. Third, low-abundance proteins or proteins with impaired function and/or conformation might be missed or undetectable by MS-based proteomics [14].

## 5 Metabonomics

Metabonomics is being increasingly used to analyze biological samples and measure the systematic, dynamic metabolic responses of organisms [49]. Metabonomics provides direct information about endogenous and exogenous metabolites, dovetailing beautifully with systems biology and allowing integration with other omics technologies [49]. Recently, metabonomics has attracted interest for cancer biomarker discovery, with implications for early diagnosis.

Hirata et al. identified candidate metabolites as PDAC biomarkers using gas chromatography/mass spectrometry (GC/MS). They reported that the combination of histidine, xylitol, and CA19-9 had 90.7% sensitivity and 89.5% specificity to detect PDAC, which they confirmed in an independent cohort [50]. Sakai and colleagues constructed a two-phase screening strategy using GC/MS and liquid chromatography/mass spectrometry (LC/MS) to detect a wide range of metabolites. When they screened mannose by GC/MS and lysophosphatidylcholine (LPC) 18-0 by LC/MS, they could detect PDAC with 100% sensitivity and 80% specificity in a training set and 84.1% sensitivity and 84.1% specificity in a validation set [51]. Similarly, Kobayashi and coworkers developed a serum metabonomics-based diagnostic model based on xylitol, 1,5-anhydro-D-glucitol, histidine, and inositol that displayed 86.0% sensitivity and 88.1% specificity to detect PDAC [52]. More recently, Unger and colleagues reported a six-metabolite biomarker panel consisting of 5-hydroxytryptophan, LysoPE (18:2), PC (16:0/16:0), PC (18:0/22:4), PE (17:0/0:0), and SM (d18:1/16:0) that had 90% sensitivity and 85% specificity to detect PDAC [53].

Those metabonomics studies were supported by recent work that proposed a “metabolic timeline” of PDAC based on principal component analysis (PCA) of 215 metabolites in patients with pancreatic neuroendocrine tumors, IPMN, localized PDAC, or advanced PDAC [54]. The authors reported that 10 metabolites were different between early-stage PDAC and IPMN, and that PCA could be a useful tool for early diagnosis of PDAC. Challenges in metabonomics include compound annotation, identification of unknown constituents, accurate measurement of metabolite abundance, and analysis of high-throughput metabonomics data [55]. Additionally, metabonomics in cancer research specifically requires robust ways to collect samples in order to precisely and effectively profile the heterogeneous tumor environment [55]. Overall, metabonomics allows researchers to identify novel biomarkers that were not previously known to be involved in PDAC carcinogenesis and development. With improvement in separation technologies and mass accuracy, we believe that metabonomics will be applied more frequently to aid disease detection in the near future.

## 6 Sampling sources in biomarker research

Body fluids such as blood, cyst fluid, pancreatic juice, bile, and urine are characteristically enriched with biomarkers that can be used for early diagnosis of PDAC. Here, we present a comprehensive overview of the sampling sources used in diagnostic biomarker studies of PDAC (Table 1).

**Table 1:** List of proteomic biomarkers for early diagnosis of pancreatic ductal adenocarcinoma.

Diagnostic biomarker	Body fluids	Expression pattern	AUC or accuracy	Sensitivity/specificity	Year	Author
SYCN	Blood	↑	0.790	NA	2013	Makawita et al. [41]
REG1B	Blood	↑	0.790	NA	2013	Makawita et al. [41]
IL-11	Blood	↑	0.901	97.7%; 70%	2014	Ren et al. [56]
MIC-1	Blood	↑	0.935	65.8%; 96.4%	2014	Wang et al. [57]
CFB	Blood	↑	0.958	90.1%; 92.7%	2014	Lee et al. [58]
C4BPA	Blood	↑	0.86	67.3%; 95.4%	2016	Sogawa et al. [42]
DTNBP1	Blood	↑	0.849	81.9%; 84.7%	2016	Guo et al. [43]
Exosomal Glypican-1	Blood	↑	1.0	100%; 100%	2015	Melo et al. [60]
Exosomal Glypican-1 and CD63	Blood	Both ↑	0.99	99%; 82%	2018	Lewis et al. [61]
Exosome-based signature	Blood	NA	0.9	90.6%; 97.1%	2018	Carmicheal et al. [62]
MUC-5AC and MUC2	Cyst fluid	Both ↑	0.97	96%; 100%	2017	Jabbar et al. [64]
MUC-5AC and PSCA	Cyst fluid	Both ↑	0.96	95%; 96%	2017	Jabbar et al. [64]
ARG2	Pancreatic juice	↑	0.729	NA	2015	Pan et al. [67]
sLR11	Bile	↑	0.89	100%; 80%	2016	Terai et al. [69]
LYVE-1, REG-1-alpha, and TFF-1	Urine	All ↑	0.89–0.92	76.9%; 89.8%	2015	Radon et al. [70]
NGAL	Urine	↑	NA	80.95%; 80%	2016	Hogendorf et al. [71]
CA19-9, IGFBP2, and IGFBP3	Serum	↑, ↑, and ↓	0.9	NA	2016	Yoneyama et al. [72]
CA19-9 and MUC5AC	Serum	Both ↑	0.84	83%; 75%	2017	Kaur et al. [47]
CA19-9 and THBS2	Serum	Both ↑	0.97	87%; 98%	2017	Kim et al. [48]
CA19-9, TIMP1, and Apo-A4	Serum	↑, ↑, and ↓	0.934	86%; 90%	2017	Park et al. [73]
CA19-9, LRG1, and TTR	Serum	↑, ↑, and ↓	0.931	82.5%; 92.1%	2017	Park et al. [74]
CA19-9, Apo-E, ITIH3, Apo -A1, and Apo-L1	Serum	↑, ↑, ↑, ↓, and ↓	0.99	95%; 94.1%	2017	Liu et al. [75]
CA19-9, TIMP-1, and LRG1	Serum	All ↑	0.949	84.9%; 65.8%	2017	Capello et al. [46]
CA19-9, TFPI, and TNC-FNIII-B	Serum	↑, ↑, and ↓	0.92	95%; 85%	2017	Balasenthil et al. [76]
CA19‐9, PROZ, and TNFRSF6B	Serum	All ↑	0.932	76.1%; 100%	2019	Wu et al. [77]
CA19‐9, TFF1, TFF2, and TFF3	Serum	All ↑	0.93	NA	2019	Jahan et al. [78]

*Note:* AUC: area under receiver-operating characteristic curves; SYCN: syncollin; REG1B: regenerating islet-derived 1 beta; IL: interleukin; MIC: macrophage inhibitory cytokine; CFB: complement factor b; C4BPA: complement component 4 binding protein α-chain; DTNBP1: dysbindin; MUC: mucin; PSCA: prostate stem-cell antigen; ARG2: anterior gradient-2; sLR11: LDL receptor-relative with 11 ligand-binding repeat; LYVE-1: lymphatic vessel endothelial hyaluronan receptor 1; REG: regenerating gene; TFF: trefoil factor; NGAL: neutrophil gelatinase-associated lipocalin; CA19-9: carbohydrate antigen 19-9; IGFBP: insulin-like growth factor-binding protein; THBS: thrombospondin; TIMP: metalloproteinase; Apo: apolipoprotein; LRG: leucine-rich alpha-2 glycoprotein; TTR: transthyretin; ITIH3: inter-alpha-trypsin inhibitor heavy chain H3; TFPI: tissue factor pathway inhibitor; TNC-FN III-C: tenascin C; PROZ: vitamin K-dependent protein Z; TNFRSF6B: tumor necrosis factor receptor superfamily member 6b; TFF: trefoil factor.

### 6.1 Blood

Blood is an easily accessible, non-invasive, and cost-effective sample source for studies of diagnostic biomarkers. A comprehensive understanding of blood can be gained using genomics, transcriptomics, proteomics, and metabonomics. Plasma interleukin-11 (IL-11) presented 97.7% sensitivity and 70% specificity as a diagnostic biomarker of PDAC [56]. Wang et al. found that macrophage inhibitory cytokine 1 (MIC-1) could serve as a novel diagnostic biomarker of PDAC, particularly in patients with early-stage disease [57]. Additionally, Lee et al. found that complement factor b (CFB) could serve as a potential diagnostic biomarker to discriminate PDAC from healthy controls, chronic pancreatitis, and other gastrointestinal cancers [58].

Exosomes are nano-sized, extracellular vesicles that are released from different types of cells and carry various pathogenic RNAs, DNAs, and proteins. Exosomes can implicate disease states and might therefore be useful as entities for non-invasive diagnostics [59]. Genomics, proteomics, and metabonomics have been used to explore the link between exosomes and cancer development [63]. Melo et al. reported that circulating glypican-1^+^ exosomes could detect early PDAC with absolute sensitivity and specificity [60]. Lewis et al. developed a bivariate model consisting of exosomal glypican-1 and CD63 to detect PDAC with 99% sensitivity and 82% specificity [61]. Carmicheal et al. used principal component differential function analysis and surface-enhanced raman spectroscopy to show that tumor-specific spectral signatures in exosomes could serve as a tool to diagnose PDAC at an early stage [62].

### 6.2 Cyst fluid

Some pancreatic cysts are precancerous or cancerous, whereas others are benign. Characterization of precursor lesions can provide new insights into early PDAC detection. Cyst fluids are rich with proteins that can be analyzed by genomics, transcriptomics, proteomics, and metabonomics. Cyst fluids can share some features of the pancreatic microenvironment and thus serve as potential biomarkers of PDAC. A review by Thiruvengadam et al. systematically discussed the most promising biomarkers in cyst fluid to distinguish high-risk cysts from low-risk cysts, including mucin-1, amphiregulin, IL-1B, SPINK1, monoclonal antibody Das-1, and miR-21 [45]. In addition, Jabbar et al. conducted a phase IIc diagnostic study using targeted MS and reported that mucin-5AC and mucin-2 in cyst fluid could discriminate premalignant/malignant pancreatic cystic lesions from benign lesions with 97% accuracy, and mucin-5AC combined with prostate stem-cell antigen (PSCA) could identify high-grade dysplasia/cancer with 96% accuracy [64]. Analysis of cyst fluid for tumor biomarkers is a useful supplement to other diagnostic methods and has the potential to improve PDAC diagnosis.

### 6.3 Pancreatic juice

Pancreatic juice is a rich source of cancer biomarkers that can be analyzed using genomics, transcriptomics, proteomics, and metabonomics. The procedure to collect pancreatic juice is invasive, however. Mateos and colleagues conducted a genomic analysis of pancreatic juice DNA (PJD) and found that the mutational burden and copy-number alterations in PJD could be used to discriminate between early invasive carcinoma and IPMN [65]. A study using NGS showed that *TP53* mutation in pancreatic juice provided a helpful biomarker to diagnosis malignant IPMN preoperatively [66]. Furthermore, miR profiling of pancreatic juice showed that the combination of miR-205, miR-210, miR-492, and miR-1427 could predict PDAC [33]. Proteomic analysis of pancreatic juice showed that overexpression of anterior gradient-2 (ARG2) was a potential biomarker for early PDAC diagnosis [67].

### 6.4 Bile

Bile is a good indicator of abnormal changes linked to pancreato-biliary malignancies. Bile can be collected during surgery or endoscopy and can be tested using genomics, transcriptomics, proteomics, and metabonomics. Advances in proteomics have made it possible to identify the complex composition of bile. Navaneethan et al. performed bile proteomics to identify markers to differentiate between malignant tumors and benign diseases [68]. Terai et al. reported that LDL receptor-relative with 11 ligand-binding repeats (sLR11) could serve as a diagnostic biomarker of PDAC and biliary tract cancer [69]. Biomarkers in bile might be able to detect PDAC earlier than some makers in blood; however, the clinical use of bile is limited because of difficulty in sampling, small sample sizes, as the fact that bile composition is affected by the metabolic function of liver.

### 6.5 Urine

Urine is an ideal source of biomarkers because it is readily available, can be obtained non-invasively, and is amenable to proteomics and metabonomics analyses. On the other hand, it contains limited amounts of protein, RNA, and DNA and has little association with the pancreas. Radon et al. conducted a study to identify urine proteins to detect early-stage PDAC and established a three-protein biomarker panel including lymphatic vessel endothelial hyaluronan receptor (LYVE)- 1, regenerating gene (REG)-1A, and trefoil factor (TFF)-1, which provided an AUC between 0.89 and 0.92 for early PDAC detection [70]. In another study, neutrophil gelatinase-associated lipocalin (NGAL) in urine was shown to be a potential diagnostic biomarker for early PDAC detection [71]. Prospective, large-sample, multi-center clinical trials are warranted to identify and verify urinary diagnostic biomarkers of PDAC.

### 6.6 Biomarker panels

Although CA19-9 is the only FDA-approved PDAC biomarker, many biomarker panels have been constructed to improve the accuracy of PDAC diagnosis (Table 1). In 2016, Yoneyama and colleagues used antibody-based proteomics and LC-MS/MS to show that the combination of CA19-9, insulin-like growth factor-binding protein (IGFBP)2, and IGFBP3 could significantly improve the accuracy of PDAC diagnosis compared with CA19-9 alone [72]. In 2017, Kaur et al. conducted a multi-center study and found that the combination of CA19-9 and MUC-5AC significantly improved the accuracy of early-stage PDAC diagnosis compared with CA-19-9 alone, providing a sensitivity of 83% and a specificity of 75% [47]. Kim et al. found that elevated levels of THBS-2 and CA19-9 could be used to discriminate between healthy individuals and individuals with early PDAC with greater sensitivity and specificity than elevated CA19-9 alone [48]. Park et al. used MS and ELISA to show that levels of apolipoprotein (Apo)-A4, Apo-C3, IGFBP2, and tissue inhibitor of metalloproteinase (TIMP) 1 were significantly changed in PDAC compared with those in pancreatitis [73]. Those authors also showed that the combination of CA19-9, Apo-A4, and TIMP1 had 86% sensitivity and 90% specificity to differentiate early PDAC from pancreatitis [73]. In another study, Park and colleagues measured 1000 marker candidates with multiple reaction monitoring-mass spectrometry (MRM-MS) and proposed a triple-biomarker panel [CA19-9, leucine-rich alpha-2 glycoprotein (LRG)-1, and transthyretin] that was superior to CA19-9 alone, providing 82.5% sensitivity and 92.1% specificity for PDAC detection [74]. Similarly, Liu et al. established a novel biomarker panel of CA19-9, Apo-E, inter-alpha-trypsin inhibitor heavy chain H3 (ITIH3), Apo-A1, and Apo-L1 that showed 95% sensitivity and 94.1% specificity for PDAC diagnosis [75]. Other three-biomarker panels consisting of CA19-9, TIMP1, and LRG1 or CA19-9, tissue factor pathway inhibitor (TFPI), and tenascin C (TNC-FN III-C) were reported to significantly improve the detection of early-stage PDAC [46], [76]. In 2019, Wu et al. analyzed 869 proteins using isobaric tags for relative and absolute quantitation (iTRAQ) and LC-MS/MS and reported vitamin K-dependent protein Z (PROZ) and tumor necrosis factor receptor superfamily member 6b (TNFRSF6B) as novel serum biomarkers for early PDAC detection. They also showed that a panel consisting of CA19-9, PROZ, and TNFRSF6B had 76.1% sensitivity and 100% specificity for early diagnosis of PDAC [77]. Jahan et al. reported that CA19-9 combined with trefoil factors (TFF1, TFF2, and TFF3) was a promising panel for discriminating early-stage PDAC from benign diseases with an AUC of 0.93 [78]. In summary, several promising biomarker panels have demonstrated high diagnostic accuracy and the ability to complement CA19-9 in early PDAC diagnosis, warranting clinical verification and validation.

## 7 Integrated omics analysis and bioinformatics

In the post-genomics era, a systems biology perspective integrating multi-omics to explore diagnostic biomarkers holds immense potential to improve early PDAC diagnosis (Fig. 1). Multi-omics studies generate great quantities of raw data. How to process and analyze those data with accuracy, consistency, and transparency and thus generate real biological insights are major challenges in multi-omics research, requiring the support of bioinformatics [79].

The Cancer Genome Atlas Research Network performed a study integrating genomics, transcriptomics, and proteomics of PDAC specimens and provided a complex characterization of PDAC with new information for early diagnosis [80]. Vandenbrouck et al. implemented a bioinformatics tool and designed a workflow to exploit the ever-increasing omics data and identify candidate biomarkers for early cancer diagnosis [81]. Long et al. utilized an integrative method along with omics-based data and supervised machine-learning techniques to identify and validate potential biomarkers, resulting in a panel of ADAM9, ANXA2, APLP2, and LAMC2 that could accurately detect PDAC in early stages [82].

Huang et al. [83] summarized the available multi-omics data integration methods, including unsupervised data integration, supervised data integration, and semi-supervised data integration. Another recent review summarized the tools and methods that can be used to integrate multi-omics data and discussed their application in explorations of diagnostic biomarkers for cancers [84]. The tools and methods discussed in that review included iClusterPlus, multi-omics factor analysis, network-based integration of multi-omics data, feature selection multiple kernel learning, and penalized multivariate analysis, which allow multi-omics data sets to be combined in order to interpret the underlying molecular features of PDAC and discover early diagnostic biomarkers [84].

The integration of data from multi-omics platforms comes with many challenges [84]. First, the underlying heterogeneity of single omics data sets presents a major challenge to multi-omics data integration. Multi-omics data are generated using various platforms, and data storage methods and formats vary considerably. Second, there has been little research on how to prioritize the various tools for the integration and analysis of multi-omics data. Additionally, there is no reliable and robust method to combine omics data with non-omics data, especially clinical information.

Each omics technology has its own disadvantages and advantages. It is often not possible to identify any single, definitive diagnostic biomarker for a given disease. High-throughput multi-omics technologies together with advanced bioinformatics have the potential to offer a brand-new paradigm for cancer biomarker research. Future studies are warranted to validate biomarkers by using bioinformatics to integrate multi-omics data.

## 8 Conclusions and perspectives

Early diagnosis and treatment of PDAC is a complicated and ongoing medical concern. For many oncologists, the identification of robust, reasonable, and reliable diagnostic biomarkers is a major goal. Most biomarker-associated studies are based on small datasets gained from one or two specific platforms and lack reasonable external validation. A systems biology perspective aims to organize multi-omics data, understand complex tumor characteristics, and identify biomarkers with high accuracy, sensitivity and comprehensiveness, which has the potential to become the norm in the near future.

Although many novel diagnostic biomarkers have been discovered through omics studies of PDAC in the past decade, none of those novel biomarkers has yet been brought into routine clinical practice. Ideally, large, prospective, externally validated studies will be conducted to form the basis for utilization of novel biomarkers in clinical practice. Novel, clinically applicable biomarkers should answer three fundamental questions in a convincing way: Can the clinician measure them? Do they add new information? Will they help the clinician to diagnose diseases [85]? Using the systems biology approach and multi-omics techniques, body fluids and tumor tissues can be easily investigated to gain a wealth of information about potential biomarkers for early cancer detection.

Accumulated studies have demonstrated that biomarker panels are more effective and accurate than single biomarkers for PDAC diagnosis. Single biomarkers, such as CA19-9, cannot provide the sensitivity and specificity required for most clinical applications, whereas panels including CA19-9 and other biomarkers can significantly increase the accuracy of diagnosis. A comparative study also demonstrated that biomarker panels with high analytical performance could add complementary value to CA19-9 in the early detection of PDAC [45].

In conclusion, it remains a major challenge to integrate multi-omics techniques and data sets and translate them into early detection. Despite the challenges, the systems biology perspective holds great promise to support and guide the exploration of novel PDAC biomarkers.

## Financial support

This work was supported by grants from the National Science Foundation for Distinguished Young Scholars of China (81625016), the 10.13039/501100001809National Natural Science Foundation of China (81872366, 81871941, 81827807, 81802675, 81701630 and 81702341), the Outstanding Academic Leader Program of the “Technological Innovation Action Plan” in the Shanghai Science and Technology Commission (18XD1401200), the Scientific Innovation Project of the Shanghai Education Committee (2019-01-07-00-07-E00057), the Natural Science Foundation of Shanghai (19ZR1410800), the Clinical and Scientific Innovation Project of the Shanghai Hospital Development Center (SHDC12018109), and the Young Talented Specialist Training Program of Shanghai. The funding agencies had no role in the study design, the data collection and analysis, the decision to publish, or the manuscript preparation.

## Previous communication of work

This work has not been previously communicated to a society or meeting.

## CRediT authorship contribution statement

**Wu-Hu Zhang:** Conceptualization, Writing - original draft preparation. **Wen-Quan Wang:** Conceptualization, Writing - original draft preparation. **Xuan Han:** Writing - reviewing & editing. **He-Li Gao:** Writing - reviewing & editing. **Tian-Jiao Li:** Writing - reviewing & editing. **Shuai-Shuai Xu:** Writing - reviewing & editing. **Shuo Li:** Supervision, Writing - reviewing & editing. **Hua-Xiang Xu:** Supervision, Writing - reviewing & editing. **Hao Li:** Supervision, Writing - reviewing & editing. **Long-Yun Ye:** Supervision, Writing - reviewing & editing. **Xuan Lin:** Supervision, Writing - reviewing & editing. **Chun-Tao Wu:** Supervision, Writing - reviewing & editing. **Jiang Long:** Supervision, Writing - reviewing & editing. **Xian-Jun Yu:** Conceptualization, Writing - original draft preparation. **Liang Liu:** Conceptualization, Writing - original draft preparation.

## Declaration of Competing Interest

The authors declare that they have no known competing financial interests or personal relationships that could have appeared to influence the work reported in this paper.
